# Type 2 Diabetes Risk and Lipid Metabolism Related to the Pleiotropic Effects of an *ABCB1* Variant: A Chinese Family-Based Cohort Study

**DOI:** 10.3390/metabo12090875

**Published:** 2022-09-16

**Authors:** Junhui Wu, Xiaowen Wang, Hongbo Chen, Ruotong Yang, Huan Yu, Yiqun Wu, Yonghua Hu

**Affiliations:** 1 Department of Epidemiology and Biostatistics, School of Public Health, Peking University, Beijing 100191, China; 2 School of Nursing, Peking University, No. 38 Xueyuan Road, Beijing 100191, China; 3 Medical Informatics Center, Peking University, Beijing 100191, China

**Keywords:** pleiotropic effects, lipid profiles, type 2 diabetes, mediation

## Abstract

The single nucleotide polymorphism (SNP) rs4148727 in *ABCB1* (encoding p-glycoprotein) is associated with lipid levels; however, its association with type 2 diabetes (T2DM) and its the genetic correlation with lipid profiles and T2DM are unclear. We included 2300 participants from 593 families. A generalized estimating equations (GEE) model and Cox regression models were used to estimate the SNP’s effects on T2DM and lipid profiles. The participation of the SNP in T2DM pathogenesis through lipid-associated pathways was tested using mediation analysis. The G allele of the SNP was related to a 32% (6–64%, *p* = 0.015) increase in T2DM risk. It was also associated with a 10% (1–20%, *p* = 0.029), 17% (3–32%, *p* = 0.015), and 4% (1–7%, *p* = 0.015) increment in total cholesterol (TC), triglyceride (TG), and apolipoprotein A (Apo-A) concentrations, respectively. According to the mediation analysis, only TG (6.9%) and Apo-B (4.0%) had slight but significant mediation effects on the total impact of the SNP on T2DM. The pleiotropic effects of the *ABCB1* variant on T2DM and lipids likely act via different pathways. The biological mechanisms should be verified in a future study.

## 1. Introduction

Globally, one of the fastest growing diseases is type 2 diabetes mellitus (T2DM) [1]. The International Diabetes Federation (IDF) estimated that in 2109, 463 million adults had diabetes worldwide, which could exceed 700 million by 2045 [2]. China has the largest number of patients with diabetes in the world, and its incidence is still increasing [3]. Type 2 diabetes can lead to serious complications, such as cardiovascular disease and neuropathy, bringing a heavy disease burden to patients and society [1].

Several large-population based studies, conducted mainly using multiple ethnicities, have reported that lipid profiles are associated with risk of diabetes [4,5,6,7,8,9]. Furthermore, a bidirectional relationship between lipid profiles and diabetes has been reported [5,6]. High levels of triglycerides reduce insulin sensitivity and islet β cell function through lipid toxicity, inflammatory responses, and enhanced endoplasmic reticulum (ER) pressure, resulting in a glucose metabolism disorder in vivo (via inflammation or ER pressure pathways) [8,10,11]. By contrast, insulin reduces the production of free fatty acids by adipocytes, inhibits the production of very low-density lipoprotein (VLDL) in liver cells, and increases the production of low-density lipoprotein (LDL) [10,12,13]. At the same time, an abnormally elevated blood glucose level will be transformed into excessive fatty acids through the lipid regeneration pathway, combining with VLDL in liver cells to form LDL, which will be secreted [10,11,12,13]. Therefore, prediabetes or diabetes is usually accompanied by dyslipidemia because of decreased insulin secretion [10,13,14]. The comorbidity between lipid profiles and T2DM remains a common public health concern.

Family-based genetic studies have reported that lipid profiles and T2DM have a certain heritability, and some studies suggest that blood lipid profiles and T2DM might share a genetic basis [8,14,15,16]. Previous studies have reported that some adiponectin genes and some genes in the obesity pathway may influence both lipid levels and type 2 diabetes [17,18]. In addition, this genetic correlation was different among different ethnic groups and previous studies were conducted in western countries [14,15,16]. However, to date, the common genetic risk factors and biological pathways between T2DM and lipid profiles have not been fully determined. Intestinal epithelial cells, hepatocytes, and renal tubules express ABCB1 (formerly MDR1), which encodes ATP binding cassette subfamily B Member 1 (also known as p-glycoprotein (P-GP)), a protein involved in lipid synthesis and reabsorption from the intestine [19,20]. ABCB1 has been reported to have relationship with lipid metabolism susceptibility [20,21]. The association between genetic variation of ABCB1 and lipid levels remained after adjustment for age, sex, body mass index, statin use and underlying ischemic heart disease [19]. However, the association between T2DM and the non-synonymous single nucleotide polymorphism (SNP) rs4148727 in *ABCB1* remains to be explored, including its regulation and mechanism of action on blood lipids and T2DM. Further exploration of the pleiotropic effect of ABCB1 on lipid metabolism and T2DM will help us to better understand the shared genetic basis of lipid metabolism and T2DM, so as to accurately prevent the co-occurrence of these two diseases.

Therefore, this study aimed to investigate the relationship between rs4148727 T2DM and lipid profiles (i.e., apolipoprotein B (APO-B), apolipoprotein A (APO-A), high-density lipoprotein (HDL) cholesterol (HDL-C), LDL cholesterol (LDL-C), total triglycerides (TG), and total cholesterol (TC)) in Chinese families, to explain ABCB1’s pleiotropic genetic effect.

## 2. Materials and Methods

### 2.1. Population and Study Design

This was a cohort study. We selected the study population from the Fangshan/Family-based Study in China (FISSIC) program. The FISSIC program was conducted in Fangshan District (39°30′~39°55′ N, 115°25′~116°15′ E), Beijing, from 2005 to 2017. The FISSIC project is a community-based, hospital-centered genetic epidemiological study of multiple chronic diseases. The study design was an ischemic stroke, type 2 diabetes, hypertension spectrum-based family study involving the proband, their siblings, and their parents. Cases diagnosed as chronic diseases are the probands; After obtaining their informed consent, their parents, siblings, and unaffected spouses were recruited and screened using the proband initiated contact method. Disease diagnosis is verified at the central hospital. Demographic data were collected through questionnaires and longitudinal follow-up was arranged. Clinical examinations were conducted through the Preventive Health Network in the study area, and blood samples were collected from all enrolled participants. Samples are sent to a central laboratory for processing, testing and genotyping. Genotypic data were then combined with clinical, environmental, and follow-up data for analysis. The proband-initiated contact method was used to recruit 8323 participants from 5727 families. All participants provided written informed consent, physical measurements, and completed a questionnaire. The study details and design have been published previously [22,23,24]. The Ethics Committee of Peking University Health Science Center approved the study (IRB00001052-13027; Beijing, China (22 July 2013)).

The participants were followed up mainly by telephone and the community health registration system, death registration system, and medical insurance claims system. Follow-up variables included height, weight, blood pressure and fasting blood glucose, as well as behavioral factors, such as smoking, alcohol consumption, and exercise. Until 2021, the current median follow-up time was 6.4 years.

In this study participants were excluded if they were diagnosed with T2DM at < 40 years old (n = 882), had prior medical histories of serious mental disease or type 1 diabetes (n = 36), were stated to have T2DM (n = 2559) based only on glucose testing at baseline or were self-reported, or had missing biological data (n = 2546). Thus, 2300 participants were analyzed in the present study. See Figure S1 for the details of study population selection. Our analysis used baseline genotypes and lipid profiles of these participants, as well as the incidence of diabetes during follow-up.

### 2.2. Definition of Phenotypes and Indicators

In the baseline survey, biochemical measurements were conducted using samples of fasting venous blood, and lipid profiles (APO-B, APO-A, HDL-C, LDL-C, TG, and TC) were collected. Qualified technicians at the Laboratory of Molecular Epidemiology in the Department of Epidemiology at Peking University measured the biochemical indexes, including lipid profiles and fasting blood glucose (FBG). The questionnaire (administered by trained staff) was used to collect basic participant characteristics such as age, sex, smoking and alcohol use (smoking and non-smoking; drinkers and non-drinkers), medical history (coronary heart disease, hypertension, diabetes, and type of diabetes), and history of medication. The physical examination measured height and weight, from which the body mass index (BMI) was calculated (weight/height^2^ (kg/m^2^)). At the follow-up stage, participants were asked to fast for at least 8 h prior to the collection of venous blood. We defined diabetes as one of the following: (I) Fasting plasma glucose (FPG) ≥ 126 mg/dL; (ii) hemoglobin (HbA1c) > 6.5%; or (iii) continued use of antidiabetic medication.

### 2.3. Genotyping

An Amplicon ThermoEx 500 PCR instrument (Amplicon, Brighton, UK) was used to amplify a genomic fragment of the *ABCB1* gene. High-throughput, rapid, and accurate genotyping of the PCR fragment was then carried out using matrix-supported laser release/ionization time-of-flight mass spectrometry (MALDI-TOF MS, San Diego, CA, USA). The analysis included a negative (blank) control and positive controls (standard reference G:G, A:G, and A:A genotypes at rs4148727). 100% reproducibility of the 5% random sample and a call rate > 95% validated the genotyping procedure.

### 2.4. Statistical Analysis

The mean ± standard deviation was used to express the continuous variables, which were compared using a paired T test. Proportions were used to describe classification variables, and groups were compared using McNemar’s chi-squared tests. Chi-squared statistics were also used to test the Hardy-Weinberg equilibrium (HWE). The HWE for rs4148727 had a *p* value of 0.156, showing no deviations for rs4148727. Then, rs4148727 was assumed to follow an additive inheritance model, and the genotypic values were encoded as the count of the G risk allele. The relationship between the number of risk alleles and disease risk was demonstrated as linear using an additivity model.

We used a generalized estimating equations (GEE) model to estimate the correlation between rs4148727 and lipid parameters. Multilevel Cox regressions accommodating a family-based design were used to calculate the hazard ratio (HR) and 95% confidence interval (CI) for the association between genotype categories and the incidence of diabetes (dichotomous outcome). Potential confounders, such as coronary heart disease, hypertension, BMI, drinking status, smoking status, sex, and age, were used to adjust all models. The results were shown as percentage change (PC) and 95% CIs per G allele of rs4148727, associated with an increased risk of T2DM and the lipid concentration, or per 1 mmol/L lipid concentration and increased risk of T2DM. Subgroup analyses were performed for smokers and non-smokers, drinkers and non-drinkers, and participants with a BMI ≥ 24 and BMI < 24, respectively (the results are shown in the supplementary data).

To evaluate whether *ABCB1* affects T2DM pathogenesis via lipid-mediated pathways, we used counterfactual-based mediation analysis. The mediation comprised two regressions. First, T2DN, as the binary outcome (T2DM), was regressed on the exposure (rs4148727), the proposed mediator (lipid parameters), and other relevant covariates. The second regression was for the lipid parameters (mediator), which were regressed on the exposure and the same covariates. The two regressions could reveal the effects mediated by lipids (indirect effect) and by pathways other than those involving the mediators (direct effect). The mediated proportion mediated was calculated using:

HR^d^ × (HR^d^ − 1)/(HR^d^ × HR^i^ − 1),

in which HR^d^ represents the direct effect odds ratio and HR^i^ represents the indirect effect odds ratio. The statistical tests were all two-tailed, and statistical significance was indicated by a *p* value < 0.05. The R Programming Language (R Foundation for Statistical Computing, Vienna, Austria) was used to carry out all the statistical analyses.

## 3. Results

### 3.1. Population Characteristics

Table 1 summarizes the basic demographic characteristics of the study population. A total of 2300 adults were assessed. After a mean follow-up of 6.4 years, 344 patients were newly diagnosed with T2DM. Compared with people without T2DM, patients with T2DM (n = 344) tended to be male and older, with higher TG and BMI, but lower HDL-C concentrations. Patients with T2DM were more likely to have coronary heart disease, while there were more smokers and drinkers among the controls.

### 3.2. Baseline Lipid Parameters and the Risk of T2DM

The risk of T2DM was associated significantly with baseline lipid parameters Apo-B, HDL-C, and TG. Increased TG and Apo-B by 1mmol /L increased the risk of T2DM by 12% (3–20%, *p* = 0.003) and 6% (5–8%, *p* = 0.008), respectively. In contrast, an increase of HDL-C of 1 mmol/L decreased the risk of T2DM by 38% ( −58% to 7%, *p* = 0.020) (Table S1).

### 3.3. Pleiotropic Effects of ABCB1 rs4148727 on T23DM and Lipid Parameters

Table 2 shows the risk of different lipid parameters and T2DM associated with rs4148727. Under the adjusted additive model, baseline Apo-A, TG, and TC were significantly and positively associated with rs4148727. Per allele-G of rs4148727 was associated with higher levels of TC, TG, and Apo-A (10% (1–20%, *p* = 0.029), 17% (3–32%, *p* = 0.015), and 4% (1–7%, *p* = 0.015, respectively).

After adjustment for the covariates, the additive model showed that per allele-G was associated significantly with an increased risk of T2DM (by 32% (6–64%, *p* = 0.015). After adding the lipid parameters as covariates, rs4148727 remained positively correlated with T2DM (32%, 5–65%, *p* = 0.015).

To further explore the pleiotropic effects of rs4148727on lipid and T2DM, we performed subgroup analysis for the effects of drinking status, smoking, and BMI. The results showed that the associations between rs4148727 and certain lipid parameters disappeared. Per allele-G of rs4148727 was associated with A 37% (8–76%, *p* = 0.011) increase in the risk of T2DM in the BMI ≥ 24 subgroup was associated with the per allele-G of rs4148727. A 46% (1–111%, *p* = 0.046) increase in the risk of T2DM in the smoker subgroup was associated with the per allele-G of rs4148727 (Table S2).

To determine whether *ABCB1* is involved directly in T2DM pathogenesis or through lipid pathways, mediation analysis was carried out. In the mediation analysis, we combined the models for T2DM and lipid parameters to determine the indirect effect explained by lipid parameters and the direct effect exerted by other pathways. The results showed that the indirect was negligible, but there was evidence of a direct effect. TG and Apo-B showed small but significant effects, mediating 6.9% and 4.0% of the total effects B, respectively (Table 3).

## 4. Discussion

In this study, we found that *ABCB1* rs4148727 was significantly associated with the lipid profile and the risk of T2DM, suggesting that rs4148727 has pleiotropic effects on lipid profile and T2DM in a Chinese population. We observed that lipid metabolism plays a mediating role in the association between rs4148727 and T2DM, complementing the evidence for a relationship between diabetes mellitus and lipid profiles.

The common genetic components of lipid profiles and T2DM have not been fully explored [16]. In this study, rs4148727 was found to increase the risk of elevated TC, TG, and APO-A levels, and was associated with an increased risk of T2DM. A study based on two large cohorts in the Netherlands (the Life Lines cohort and the PREVEND cohort) found that 15 lipid loci showed a pleiotropic association with glucose traits at the SNP level, suggesting complex genetic regulation and metabolic interplay between lipids and glucose [16]. A prospective study of 3474 people without diabetes found that during 5 years of follow-up, a larger increase in fasting serum TG levels in nondiabetic individuals was associated with a higher genetic risk load and increased insulin resistance enhanced this effect [25]. A study in India found that lipids and blood sugar traits were related; however, the results based on genetic variants found that only TG caused a 1.15% variation in the homeostatic model assessment for insulin resistance (HOMA-IR), 1.53% in the homeostasis model assessment of β-cell function (HOMA-β), and 1.18% in fasting insulin [26]. However, there is also inconsistent evidence. Another study based on the Danish Inter99 cohort found that individuals with alleles associated with elevated TG indeed had elevated TG levels but no increased risk of developing T2DM during a five-year follow-up [27]. More evidence is needed to determine the shared genetic mechanism between lipid profiles and T2DMs.

Although the underlying mechanism of the association between rs4148727, lipid profiles, and T2DM has not been reported, indirect evidence has been found in previous studies. P-glycoprotein (P-gp, encoded by *ABCB1*) is an efflux pump expressed by renal tubular cells, hepatocytes, and intestinal epithelial cells. Using mice, P-gp was suggested to be involved in the reabsorption of cholesterol from the intestines [19]. Moreover, cholesterol biosynthesis was blocked in cell lines with inhibited P-gp activity [28]. In addition, plasma lipid levels are associated with polymorphisms in human *ABCB1* in both men and women [29,30,31,32]. *ABCB1* contains four SNPs (rs4148727, rs3213619, rs1128503, rs3842), among which rs3842 has been suggested by genome-wide association study analysis to be a susceptibility gene locus for T2DM. In a 2012 Korean study of 121 patients with ischemic stroke and 291 controls, *ABCB1* polymorphisms were associated with dyslipidemia (rs1128503, *p* < 0.05), diabetes mellitus (rs3842, *p* < 0.05), and ischemic stroke (rs4148727, *p* < 0.05) [33]. To the best of our knowledge, the present study was the first to find the association between rs4148727 and T2DM. Many genomic regions exert pleiotropic effects onT2DM and lipids; therefore, more research is required to determine the clusters of genes, variants, pathways, and actionable targets.

The correlation betweenrs4148727 and lipid parameters was found to be different in different subgroups. In BMI < 24 individuals, the correlation between rs4148727 and lipid parameters disappeared, which might have resulted from an insufficient sample size and lower statistical power. However, in participants with BMI ≥ 24, rs4148727’s associations with TG, TC, and T2DM remained stable. Previous studies have suggested that the function of *ABCB1* might be affected by obesity, suggesting a future direction for the exploration of ABCB1 function [34]. The correlation between rs4148727 and lipid parameters was higher in non-smokers and alcohol non-consumers than in smokers and alcohol consumers. Probably because of the publicity and popularization of community health knowledge, smoking and drinking, as recognized risk factors for cancer and cardiovascular disease, are considered as bad lifestyle habits by community residents. These subjects actively avoided smoking and drinking during follow-up, increasing health monitoring, and as a result, smokers and drinkers had lower blood lipid levels. The underlying mechanism remains to be explored. In addition, the association between rs4148727 and T2DM remained stable in patients with BMI ≥ 24 and smokers. Clinical studies have shown that tobacco smoke and the existence of polymorphisms in the *ABCB1* gene can be risk factors conferring susceptibility to certain chronic diseases [35]. Therefore, findings might be clinically useful for disease prevention, and suggest that people with the risk allele should control their weight and stop smoking.

The family-based design was the main strength of this study, which attenuated population stratification by offering similar environmental exposure. At the same time the family-based design can better integrate the results of the linkage and association analyses. The family-based design may also cause some problems, such as difficulty in recruiting subjects, especially for late-onset diseases. In addition, the similar genetic backgrounds of affected and unaffected members of a family may lead to overmatching, increasing the sample size.

However, the study had limitations. First, the population of this study is mainly representative of rural areas in northern China. A larger and more representative population is needed to further verify the results of this study. Second, the confounding factors suggested by previous research were adjusted in the data analysis of the study; however, there might still be unknown confounding factors causing bias. Third, considering that it is difficult to collect subjects in the family lineage study, the sample size of this study is limited, and more samples need to be collected in the future to reinforce the statistical robustness of this study. In addition, due to the lack of previous relevant studies, our exploration of the biological mechanism of pleiotropy is limited, further studies on disease mechanisms are needed to provide evidence for potential molecular and biological pathways of gene pleiotropy.

## 5. Conclusions

In summary, we found that varying lipid profiles and T2DM risk effects were related to the pleiotropic effects of *ABCB1* SNP rs4148727, and smokers and individuals with a BMI ≥ 24 are more vulnerable to the effects. The results provided evidence of genetic predispositions to T2DM and varying lipid profiles.

## Figures and Tables

**Table 1:** Baseline characteristics of newly diagnosed patients with type 2 diabetes (T2DM).

Characteristics	Total Participants	T2DM Cases	Non-T2DM	*p* Value
number	2300	344	1956	
Age, mean (SD), years	58.41 ± 8.94	59.09 ± 9.02	58.29 ± 8.92	0.137
Female sex, n (%)	1185 (51.5)	118 (34.3)	927 (47.4)	0.014
BMI, mean (SD), kg/m^2^	26.02 ± 3.75	26.86 ± 3.88	25.87 ± 3.71	<0.001
TC (mmol/L)	3.00 ± 0.95	3.02 ± 1.03	3.00 ± 0.93	0.684
TG (mmol/L)	1.39 ± 1.32	1.68 ± 1.88	1.34 ± 1.19	<0.001
LDL-C (mmol/L)	2.04 ± 0.75	2.06 ± 0.79	2.04 ± 0.75	0.601
HDL-C (mmol/L)	0.90 ± 0.32	0.86 ± 0.30	0.91 ± 0.32	0.009
Apo-A (mmol/L)	1.09 ± 0.32	1.09 ± 0.33	1.09 ± 0.32	0.940
Apo-B (mmol/L)	0.71 ± 0.24	0.71 ± 0.26	0.70 ± 0.24	0.506
Smoker, n (%)	1183 (47.7)	140 (40.7)	943 (48.2)	0.013
Alcohol consumer, n (%)	985 (43.5)	130 (37.8)	885 (45.2)	0.043
Hypertension (%)	443 (19.5)	65 (18.9)	378 (19.3)	0.503
Coronary heart disease (%)	224 (9.7)	64 (18.6)	160 (8.2)	<0.001
No. of rs4148727 G alleles (%)				
0	1720 (74.8)	237 (68.9)	1483 (75.8)	0.004
1	558 (24.3)	106 (30.8)	452 (23.1)	
2	22 (1.0)	1 (0.2)	21 (1.1)	

BMI: body mass index; TC: total cholesterol; TG: triglyceride; LDL-C: low-density lipoprotein cholesterol; HDL-C: high-density lipoprotein cholesterol; Apo-A: apolipoprotein A; Apo-B: apolipoprotein B. Notes: The mean ± standard deviation was used to report continuous variables. McNemar’s chi-squared test and a paired *t*-test were used to analyze the differences between controls and cases.

**Table 2:** The association of *ABCB1* rs4148727 with baseline incident T2DM and lipid parameters.

Variables	β (SE)	PC (%) (95% CI)	*p* Value
Lipid parameters			
TC	0.097 (0.045)	10.19 (0.88–20.35)	0.029
TG	0.154 (0.063)	16.65 (3.10–31.98)	0.015
LDL-C	0.053 (0.035)	5.44 (−1.55–12.93)	0.133
HDL-C	0.015 (0.015)	1.51 (−1.43–4.54)	0.313
Apo-A	0.037 (0.015)	3.77 (0.76–6.87)	0.015
Apo-B	0.007 (0.011)	0.70 (−1.45–2.90)	0.542
T2DM			
model1	0.274 (0.112)	31.52 (5.60–63.81)	0.015
model2	0.276 (0.114)	31.79 (5.40–64.78)	0.015

β: estimate; SE: standard error; PC: percentage change; CI: confidence interval; TC: total cholesterol; TG: triglyceride; LDL-C: low-density lipoprotein cholesterol; HDL-C: high-density lipoprotein cholesterol; Apo-A: apolipoprotein A; Apo-B: apolipoprotein B; T2DM: type 2 diabetes mellitus. Notes: Adjustments for body mass index, coronary heart disease, hypertension, drinking status, smoking, sex, and age and the lipid parameter model. Adjustments for HDL-C. LDL-C, TG, TC, body mass index, coronary heart disease, hypertension, drinking status, smoking, sex, and age were made for T2DM model 2.

**Table 3:** Mediation analysis of the association between the ABCB1 rs4148727 and T2DM risk mediated by lipid parameters.

Variables	Direct Effect HR (95%CI)	Indirect Effect HR (95%CI)	Total Effect HR (95%CI)	PM (%)
TC	1.40 (1.13–1.67)	0.99 (0.84–1.07)	1.40 (1.13–1.67)	0
TG	1.30 (1.04–1.66)	1.09 (1.04–1.15)	1.40 (1.13–1.68)	6.9
LDL-C	1.35 (1.09–1.71)	1.05 (0.99–1.10)	1.40 (1.13–1.67)	0
HDL-C	1.36 (1.10–1.72)	1.04 (0.98–1.09)	1.40 (1.13–1.67)	0
Apo-A	1.40 (1.14–1.76)	1.00 (0.98–1.02)	1.40 (1.13–1.67)	0
Apo-B	1.34 (1.08–1.70)	1.06 (1.03–1.09)	1.40 (1.13–1.68)	4.0

OR: odds ratio; CI: confidence interval; PM: proportion mediated; T2DM: type 2 diabetes mellitus; TC: total cholesterol; TG: triglyceride; LDL-C: low-density lipoprotein cholesterol; HDL-C: high-density lipoprotein cholesterol; Apo-A: apolipoprotein A; Apo-B: apolipoprotein B. Notes: all models were adjusted for age, sex, smoking and drinking status, hypertension, coronary heart disease, and body mass index. Lipid parameters with significant indirect effects are shown in bold.

## Data Availability

The data presented in this study are available on request from the corresponding author. The data are not publicly available due to ethical issues.

## Supplementary Materials

The following are available online at https://www.mdpi.com/article/10.3390/metabo12090875/s1, Figure S1: The flow chart of the study population selection. Table S1: Baseline Lipid Parameters and the risk of T2DM. Table S2: HRs (95% CIs) for different lipid parameters and incident T2DM by ABCB1 rs4148727. Figure S2: The original recruitment procedures in the supplement files. Ref [36] is cited in Supplementary Materials.
