# Supplementary material for: Type 2 Diabetes Risk and Lipid Metabolism Related to the Pleiotropic Effects of an ABCB1 Variant: A Chinese Family-Based Cohort Study
Source: Metabolites. 2022 Sep 16;12(9):875. doi: 10.3390/metabo12090875 (PMC9502507; doi:10.3390/metabo12090875)
Supplement: Supplementary file 1 [file metabolites-12-00875-s001.zip › metabolites-1883136-supplementary.pdf]

Supplementary Files

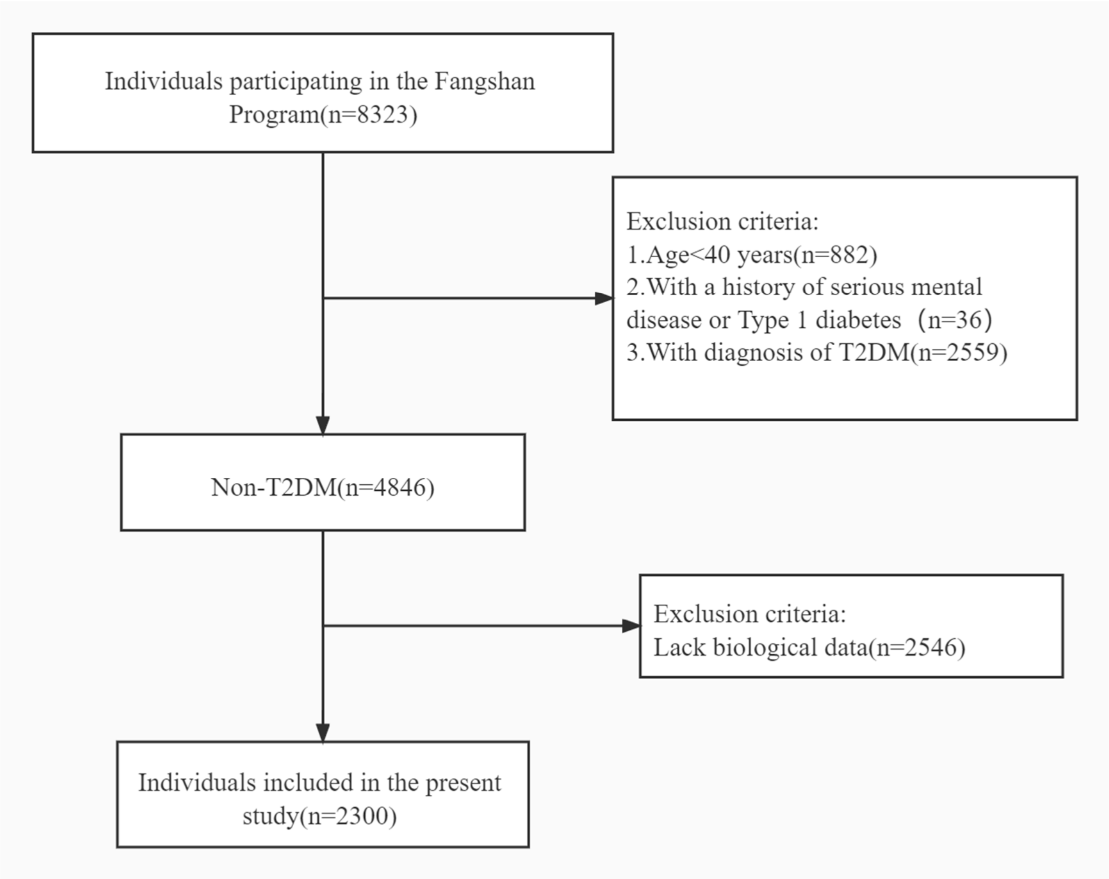

Figure S1. The flow chart of the study population selection.

Table S1. Baseline Lipid Parameters and the risk of T2DM.

Variables	β(SE)	PC (%) (95% CI)	P value
TC	0.017(0.065)	1.72(-10.45-15.54)	0.796
TG	0.110(0.038)	11.63(3.42-20.26)	0.003
LDL-C	-0.021(0.082)	-2.08(-16.62-15.00)	0.796
HDL-C	-0.480(0.206)	-38.12(-58.68--7.34)	0.020
Apo-A	0.120(0.191)	12.75(-22.46-63.95)	0.529
Apo-B	0.061(0.006)	6.29(5.05-7.53)	0.008

β: estimate; SE: standard error; PC: percentage change; CI: confidence interval; TC: total cholesterol; TG: triglyceride; LDL-C: low-density lipoprotein cholesterol; HDL-C: high-density lipoprotein cholesterol; Apo-A: apolipoprotein A; Apo-B: apolipoprotein B; T2DM: type 2 diabetes mellitus. Notes: All models were adjusted for age, sex, smoking and drinking status, hypertension, coronary heart disease, and body mass index. P values <0.05 were shown in bold.

Table S2. HRs (95% CIs) for different lipid parameters and incident T2DM by ABCB1 rs4148727.

Variables	β(SE)	PC (%) (95% CI)	P value
**BMI<24(n=702)**			
Lipid parameters			
TC	0.045(0.081)	4.60(-10.75-22.60)	0.575
TG	-0.061(0.073)	-5.92(-18.46-8.55)	0.399
LDL-C	0.015(0.066)	1.51(-10.81-15.53)	0.820
HDL-C	0.045(0.030)	4.60(-1.37-10.94)	0.140
Apo-A	0.039(0.030)	3.98(-1.96-10.27)	0.196
Apo-B	0.006(0.021)	0.60(-3.46-4.83)	0.768
T2DM	0.099(0.256)	10.41(-33.15-82.35)	0.698
**BMI≥24(n=1598)**			
Lipid parameters			
TC	0.123(0.053)	13.088(1.93-25.47)	0.022
TG	0.246(0.084)	27.890(8.48-50.78)	0.003
LDL-C	0.076(0.042)	7.896(-0.63-17.15)	0.071
HDL-C	0.010(0.017)	0.000(-3.28-3.39)	0.993
Apo-A	0.035(0.018)	3.562(-0.03-7.28)	0.047
Apo-B	0.010(0.013)	1.005(-1.54-3.61)	0.467
T2DM	0.321(0.126)	37.851(7.69-76.47)	0.011
**Non-smoke(n=1188)**			
Lipid parameters			
TC	0.153(0.062)	16.532(3.20-31.59)	0.014
TG	0.213(0.083)	23.738(5.16-45.60)	0.010
LDL-C	0.087(0.049)	9.090(-0.90-20.09)	0.077
HDL-C	0.028(0.021)	2.840(-1.31-7.16)	0.169
Apo-A	0.049(0.021)	5.022(0.79-9.44)	0.020
Apo-B	0.015(0.016)	1.511(-1.62-4.75)	0.349
T2DM	0.230(0.142)	25.860(-4.72-66.25)	0.106
**Smoke(n=1083)**			
Lipid parameters			
TC	0.035(0.064)	3.562(-8.65-17.40)	0.587
TG	0.108(0.095)	11.405(-7.52-34.21)	0.255
LDL-C	0.017(0.051)	1.715(-7.96-12.41)	0.744
HDL-C	-0.004(0.022)	-0.399(-4.60-3.99)	0.484
Apo-A	0.021(0.022)	2.122(-2.19-6.62)	0.358
Apo-B	-0.002(0.016)	-0.200(-3.28-2.98)	0.889
T2DM	0.376(0.188)	45.645(0.76-110.54)	0.046
**Non-drink(n=1280)**			
Lipid parameters			
TC	0.117(0.058)	12.412(0.33-25.95)	0.044
TG	0.090(0.070)	9.417(-4.61-25.51)	0.197
LDL-C	0.058(0.048)	5.971(-3.54-16.43)	0.222
HDL-C	0.028(0.019)	2.840(-0.92-6.74)	0.149

Apo-A	0.047(0.020)	4.812(0.78-9.01)	0.021
Apo-B	0.009(0.015)	0.904(-2.02-3.92)	0.567
T2DM	0.275(0.143)	31.653(-0.53-74.24)	0.054
**Drink(n=985)**			
Lipid parameters			
TC	0.070(0.069)	7.251(-6.32-22.78)	0.312
TG	0.240(0.113)	27.125(1.87-58.64)	0.034
LDL-C	0.047(0.053)	4.812(-5.53-16.29)	0.375
HDL-C	-0.004(0.024)	-0.399(-4.98-4.40)	0.878
Apo-A	0.023(0.023)	2.327(-2.18-7.05)	0.331
Apo-B	0.005(0.017)	0.501(-2.79-3.91)	0.784
T2DM	0.293(0.187)	34.044(-7.09-93.39)	0.119

β, estimate; SE, standard error; PC, percentage change; CI, confidence interval; TC, total cholesterol; TG, triglyceride; HDL-C, high density lipoprotein cholesterol; LDL-C, low density lipoprotein cholesterol; Apo-A, apolipoprotein-A; Apo-B, apolipoprotein-B; T2DM, type 2 diabetes mellites.

Notes: All models were adjusted for age, sex, smoking or drinking status, hypertension, coronary heart disease, and body mass index. P values<0.05 were shown in bold

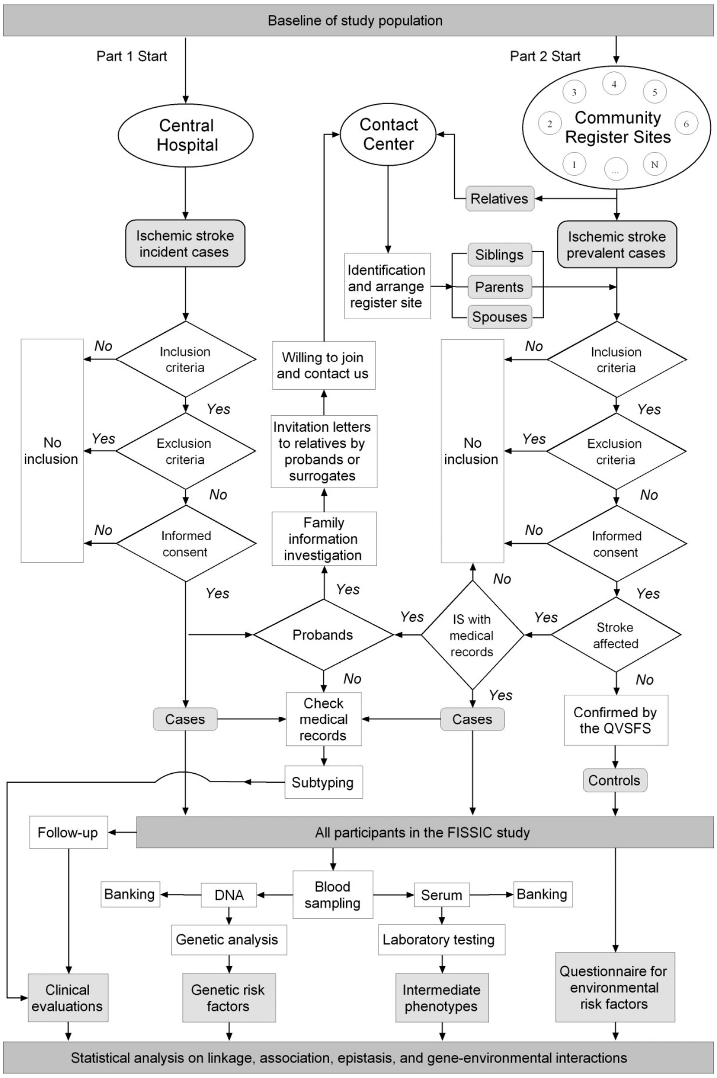

Figure S2. The original recruitment procedures in the supplement files

Tang, X., Hu, Y., Chen, D. et al. The Fangshan/Family-based Ischemic Stroke Study In China (FISSIC) protocol. BMC Med Genet 8, 60 (2007). https://doi.org/10.1186/1471-2350-8-60
